# Cognition and Behaviour in Sotos Syndrome: A Systematic Review

**DOI:** 10.1371/journal.pone.0149189

**Published:** 2016-02-12

**Authors:** Chloe Lane, Elizabeth Milne, Megan Freeth

**Affiliations:** Department of Psychology, University of Sheffield, Western Bank, Sheffield, United Kingdom; CNRS UMR7275, FRANCE

## Abstract

**Background:**

Research investigating cognition and behaviour in Sotos syndrome has been sporadic and to date, there is no published overview of study findings.

**Method:**

A systematic review of all published literature (1964–2015) presenting empirical data on cognition and behaviour in Sotos syndrome. Thirty four journal articles met inclusion criteria. Within this literature, data relating to cognition and/or behaviour in 247 individuals with a diagnosis of Sotos syndrome were reported. Ten papers reported group data on cognition and/or behaviour. The remaining papers employed a case study design.

**Results:**

Intelligence quotient (IQ) scores were reported in twenty five studies. Intellectual disability (IQ < 70) or borderline intellectual functioning (IQ 70–84) was present in the vast majority of individuals with Sotos syndrome. Seven studies reported performance on subscales of intelligence tests. Data from these studies indicate that verbal IQ scores are consistently higher than performance IQ scores. Fourteen papers provided data on behavioural features of individuals with Sotos syndrome. Key themes that emerged in the behavioural literature were overlap with ASD, ADHD, anxiety and high prevalence of aggression/tantrums.

**Conclusion:**

Although a range of studies have provided insight into cognition and behaviour in Sotos syndrome, specific profiles have not yet been fully specified. Recommendations for future research are provided.

## Introduction

Sotos syndrome is a congenital overgrowth disorder with an incidence of approximately 1 in 14,000 live births [1]. The syndrome was first recognised by Sotos et al. [2] who observed five patients with similar clinical features. These included excessively rapid growth, acromegalic features and a non-progressive cerebral disorder with mental retardation. The authors considered this combination of features to be attributable to a specific syndrome. Excessively rapid growth has been defined as advanced height, weight and bone age; acromegalic features include a prominent forehead, high anterior hairline, prominent chin and downslanting palpebral fissures [3]. Subsequent research confirmed these cardinal features in larger samples of individuals with Sotos syndrome [4,5]. As macrocephaly is one of the features of the syndrome, initial research often used the terms cerebral gigantism and Sotos syndrome interchangeably to refer to the same condition.

Until 2002, diagnosis was based on clinical assessment. The four major diagnostic criteria were confirmed by Cole & Hughes [4] in a sample of 41 typical cases. These were overgrowth with advanced bone age, macrocephaly, characteristic facial appearance and intellectual disability. Other health problems that are commonly experienced in children with Sotos syndrome are cardiac and genitourinary anomalies, neonatal jaundice, neonatal hypotonia, seizures and scoliosis [1,6]. As the syndrome is not specifically linked to the X or Y chromosomes, it affects males and females equally.

The identification of a genetic mutation responsible for Sotos syndrome was first established in a Japanese population [7]. The authors identified that haploinsufficiency of the NSD1 gene was present in a number of participants with a clinical diagnosis of Sotos. Specifically, this was a 5q35 microdeletion of the NSD1 gene which is the most common cause of Sotos in the Japanese population [8]. In individuals of non-Japanese ethnicity, an intragenic mutation of the NSD1 gene is the most common cause of Sotos, accounting for approximately 83% of cases [8].

It has since been suggested that mutations of the NSD1 gene are responsible for approximately 90% of cases of Sotos syndrome [9–11]. The remaining individuals who meet the clinical criteria but do not have an NSD1 mutation are given the diagnosis of Sotos-like or Sotos syndrome-2. Research investigating Sotos-like individuals (those who do not have an NSD1 abnormality) has suggested that haploinsufficiency of the NFIX protein could be the cause of Sotos-like features [12–15]. The authors have recognised these symptoms as a distinct overgrowth disorder which has been termed Malan syndrome.

In NSD1-positive individuals, research has investigated specific genotype-phenotype correlations associated with the different NSD1 abnormalities [16–18]. Broadly, it has been suggested that individuals with 5q35 microdeletions of the NSD1 gene have less prominent overgrowth and more severe intellectual disability, compared to individuals with mutations of the same gene [17,18]. As the genetic abnormality is not present in approximately 10% of cases, clinical assessment is still an important part of the diagnostic process.

The purpose of this review was to synthesise and critically evaluate all published literature providing data on cognition and behaviour in individuals with Sotos syndrome in order to establish current understanding of these facets of the syndrome. The specific research questions were to establish: 1) the degree of intellectual disability in individuals with Sotos syndrome; 2) whether there is evidence for a profile of verbal and non-verbal cognitive abilities; 3) whether there are common behavioural problems associated with Sotos. Behavioural problems included psychiatric and psychological issues, as well as problems with temperament.

The quality of the published research in these areas was assessed using an objective assessment tool [19]. This was important for evaluating the reliability and validity of findings within the literature. As no systematic review or meta-analysis has been published in the Sotos literature to date, this review provides a novel and comprehensive overview of the current knowledge base of the disorder. Furthermore, this review aims to identify current gaps in knowledge and suggest potential areas of interest for future research, in order to ensure that research is designed to advance areas where understanding is limited.

## Method

The review was written in accordance with the Preferred Reporting Items for Systematic Reviews and Meta-Analyses (PRSIMA) Checklist [20].

### Search Strategy

Four electronic databases were systematically searched for relevant studies: Web of Science (1964–2015), Scopus (1964–2015), PsycINFO (1964–2015) and PubMed (1964–2015). The first paper to recognise Sotos as a specific syndrome was published in 1964, so the searches were started from this date. The databases were searched using the terms “Sotos” AND “syndrome”, OR “cerebral” AND “gigantism”. The terms ‘Sotos syndrome’ and ‘cerebral gigantism’ have been used interchangeably within the literature so both were included in the database search.

In Scopus and Web of Science, the title/abstract/keywords of the journal articles were searched using the key search terms. In PsycINFO, the abstract/title/key concepts were searched and in PubMed, the title/abstract were searched. Differences in the search strategies implemented were due to the unique search system of each database. The search was conducted in August 2015. In addition to the database search, bibliographies and citations of all papers included in the review were hand-searched to ensure that all relevant papers had been identified.

### Study Selection

Predetermined inclusion criteria were used to assess whether the articles identified in the initial search were relevant. As an aim of this review was to provide an overview of findings from published research, only articles published in peer reviewed journals and written in English language were included in the review. In addition, only primary research was included in order to ensure that the same methodology and findings were not reviewed multiple times. Finally, the study was required to provide data relating to cognitive ability and/or behaviour in an individual or individuals with a diagnosis of Sotos syndrome.

When screening the abstracts, papers were considered relevant if they included the term ‘intelligence’ or if they included terms relating to specific aspects of cognition, such as ‘language’, ‘memory’, ‘attention’, ‘executive function’ or ‘logic/problem-solving’. Abstracts were also considered relevant if they mentioned any behavioural or psychiatric problems, such as ‘ASD’, ‘ADHD’, ‘psychosis’, ‘anxiety’ or ‘aggression/tantrums’. Full text articles that met all inclusion criteria were then selected for the review.

### Data Extraction

Data were extracted from articles that met inclusion criteria. This information included sample size (number of participants with Sotos), demographic information (age and gender), cognitive or behavioural assessments used and key findings from these measures. In order to satisfy the key aims of this review, studies that reported IQ scores of individuals with Sotos are summarised in Table 1; studies that reported findings related to language abilities and other specific cognitive abilities of individuals with Sotos are summarised in Table 2; studies providing data on aggression and/or tantrums in individuals with Sotos are summarised in Table 3; studies reporting findings related to ASD are summarised in Table 4; studies measuring ADHD are summarised in Table 5 and studies providing data on anxiety are summarised in Table 6.

**Table 1:** Summary of studies measuring IQ scores in Sotos syndrome (n = 25).

Author, country of study, year of publication	Sample size (n)	Gender	Mean age in years, months (range)	Cognitive assessment	Findings	Quality score (0–10)
Bale et al., USA, (1985)	3	3 (F)	(7y – 35y)	Wechsler Intelligence Scale for Children-Revised (WISC-R); Bayley Scales of Infant Development; Wechsler Adult Intelligence Scale (WAIS).	Case 1: WISC-R full scale IQ = 91, verbal IQ = 103, performance IQ = 87. Case 2: at 15 months, functional age on the Bayley cognitive scale was on the 9 month level. Developmental quotient = 61. Case 3: no developmental delay noted as a child. At age 30, WAIS full scale IQ score = 110, verbal IQ = 122, performance IQ = 93.	7.5
Bloom et al., USA, (1983)	10	7 (M), 3 (F)	Not recorded* (1y – 13y 6m)	Bayley Scales of Infant Development; Cattell Infant Intelligence Scale; Stanford Binet Intelligence Scale, Form L-M; Leiter International Performance Scale, Arthur Adaptation; Wechsler Intelligence Scale for Children-Revised (WISC-R).	Longitudinal study reporting findings from different assessments administered between the ages of 1:11 and 13:6. 6 participants had one follow-up assessment and 2 had two follow-up assessments. Full scale IQ scores ranged from 59–113.	7.5
Compton et al., USA, (2004)	1	1 (M)	20y	Wechsler Abbreviated Scale of Intelligence (WASI).	Full scale IQ = 94, verbal IQ = 100, performance IQ = 88.	7.5
de Boer et al., Netherlands, (2006)	21**	Not recorded*	Not recorded*	Dutch adaptations of Wechsler Preschool and Primary Scale of Intelligence-Revised (WPPSI-R); Wechsler Intelligence Scale for Children-Revised (WISC-R); Wechsler Adult Intelligence Scale (WAIS).	Mean full scale IQ of 76 (SD = 16), mean verbal IQ of 79 (SD = 14) and mean performance IQ of 77 (SD = 18). No significant difference between IQ scores of NSD1 mutation and NSD1 non-mutation patients.	8.6
Fickie et al., USA, (2011)	1***	1 (F)	63y	Wechsler Adult Intelligence Scale (WAIS-III).	Full scale IQ = 78.	8.3
Finegan et al., UK, (1994)	27	14 (M), 13 (F)	9y 3m (5y – 16y)	Age-appropriate versions of the UK adaptations of the Wechsler scales; Full Scale IQ (FSIQ) estimated from a short form.	IQ scores ranged from 21–103. 6 participants had an IQ < 70.	9.5
Ginter & Scott, (1975)	2	1 (M), 1 (F)	(13y 9m – 27y)	Wechsler Adult Intelligence Scale (WAIS); Wechsler Memory Scale; Bender-Gestalt Test.	Case 1: WAIS full scale IQ = 85, verbal IQ = 96, performance IQ = 72. Wechsler memory quotient = 79. Bender-Gestalt standard error score = 79. Case 2: psychometric testing indicated an IQ of 81.	4.2
Horikoshi et al., Japan, (2006)	3***	3 (M)	(2y – 3y 6m)	Enjouji Developmental Scale for Japanese Children.	Case 1: developmental quotient = 34. Case 2: developmental quotient = 66. Case 3: developmental quotient = 48.	5.1
Jung & Martin, US Virgin Islands, (1969)	1	1 (F)	8y	Wechsler Intelligence Scale for Children (WISC); Draw a Person Test; Bender-Gestalt Test; Grey-Standardised Oral Reading Paragraph Test.	WISC full scale IQ = 69. Draw a person IQ score = 64. Bender-Gestalt score corresponded with her IQ. Reading and arithmetic tests revealed functioning at the beginning first-grader level.	6.7
Leventopoulos et al., Greece, (2009)	19	9 (M), 10 (F)	2y 7m (2m – 12y)	Developmental assessment.	Developmental delay present in 16 participants. Severe mental retardation (IQ < 50) present in 13 participants.	6.8
Mauceri et al., Italy, (2000)	6	5 (M), 1 (F)	(2y – 12y)	Wechsler Intelligence Scale for Children-Revised (WISC-R); Brunet-Lezine Test.	Case 1: WISC-R IQ = 68. Case 2: WISC-R IQ = 40. Case 3: Brunet-Lezine IQ = 46. Case 4: WISC-R verbal IQ = 44. Difficulty with maths. Case 5: WISC-R IQ = 70. Case 6: IQ = 48.	6.7
Mouridsen & Hansen, Denmark, (2002)	2	2 (M)	(3y 4m – 13y)	Bayley Scales of Infant Development; Subtests from Snijders-Oomen Non-Verbal Intelligence Scale for Young Children; Wechsler Intelligence Scale for Children (WISC).	Case 1: moderate mental retardation. Case 2: WISC verbal IQ = 88, performance IQ = 78.	5.8
Okamoto et al., Japan, (2010)	1***	1 (M)	14y	Kyoto Scale of Psychological Development.	Severe mental retardation. Kyoto scale IQ score below 10.	7.5
Patterson et al., USA, (1978)	3	2 (M), 1 (F)	(6y – 10y 8m)	Stanford Binet Intelligence Scale, Form L-M; Leiter International Performance Scale; Wechsler Intelligence Scale for Children-Revised (WISC-R).	Case 1: Stanford Binet IQ = 76. Leiter IQ = 108. Case 2: WISC-R full scale IQ = 75, verbal IQ = 73, performance IQ = 72. Stanford Binet IQ = 90. Case 3: WISC-R full scale IQ = 100, verbal IQ = 100, performance IQ = 101. Stanford Binet IQ = 99.	7.5
Poznanski & Stephenson, USA, (1967)	1	1 (M)	5y 10m	Cattell Intelligence Scale; Stanford Binet Intelligence Scale.	At age 2:6 years, Cattell IQ = 60. At 5:10 years, Stanford Binet IQ = 44.	4.4
Rutter & Cole, UK, (1991)	15	Not recorded*	Not recorded*	Wechsler Intelligence Scale for Children-Revised (WISC-R); Wechsler Preschool and Primary Scale of Intelligence (WPPSI).	87% of participants completed the WISC-R and 13% completed the WPPSI. Full scale IQs ranged from 54–96 (mean = 73.8). Verbal IQs ranged from 47–102 (mean = 76.93) and performance IQs ranged from 51–101 (mean = 74.6)	6.8
Sarimski, Germany, (2003)	27	17 (M), 10 (F)	10y 7m (6y – 15y)	Parental Report; Heidelerger-Kompetenz-Inventar (HKI).	In the mild impairment group (n = 16), mean cognitive competence = 185. In the moderate impairment group (n = 11), mean cognitive competence = 153.6.	9.5
Scarpa et al., Italy, (1994)	2	1 (M), 1 (F)	(5y – 7y)	Wechsler Preschool and Primary Scale of Intelligence (WPPSI); Brunet-Lezine Test.	Case 1: WPPSI IQ = 58. Case 2: Brunet-Lezine IQ = 45.	5.8
Sobel, USA, (1995)	1	1 (F)	8y	Cattell Intelligence Scale; Vineland Social Maturity Scale.	At 22 months of age, she was delayed by approximately 4 months in mental and social age. On examination at 8 years of age, she was of normal intelligence.	5
Sotos et al., USA, (1964)	5	3 (M), 2 (F)	(2y – 11y 6m)	Stanford Binet Intelligence Scale; Clinical Observation.	Case 1: Stanford Binet IQ = 70. Case 2: Stanford Binet IQ = 70. Case 3: Stanford Binet IQ = 72. Case 4: intelligence judged to be borderline. Case 5: several months retarded in mental development.	7.5
Tei et al., Japan, (2006)	3***	2 (M), 1 (F)	(3y 4m – 37y)	Wechsler Intelligence Scale for Children (WISC-III); Development Test (New-K Style for the Japanese).	Case 1: WISC full scale IQ = 70. Case 2: development test was in the normal limit. DQ = 85. Case 3: no intelligence test performed. Had graduated from a regular senior high school with lower achievement.	7.5
Trad et al., USA, (1991)	1	1 (F)	3y 11m	Stanford Binet Intelligence Scale.	Stanford Binet IQ = 88.	6.7
Varley & Crnic, USA, (1984)	11	6 (M), 5 (F)	9y 5m (5y 11m – 13y 11m)	Wechsler Intelligence Scale for Children-Revised (WISC-R); Stanford Binet Intelligence Scale; Bayley Mental Scale.	Each participant was administered one of the cognitive assessments. 54% completed the WISC-R, 28% the Stanford Binet Intelligence Scale and 18% the Bayley Mental Scale. IQ scores ranged from 40–85 with a median of 62.	8.1
Villaverde et al., USA, (1971)	2	2 (M)	(9y – 13y 7m)	Stanford Binet Intelligence Scale; Columbia Mental Maturity Scale; Vineland Social Maturity Scale.	Case 1: at 3 years of age, Stanford Binet IQ = 56. At 9 years of age, Stanford Binet IQ = 56. Mental age measured by the Columbia Mental Maturity Scale = 3:8. On the Vineland Social Maturity Scale, SQ = 49. Case 2: Stanford Binet IQ = 48. Vineland Social Maturity SQ = 67.	8.3
Zechner et al., Germany, (2009)	3***	1 (M), 2 (F)	(8y 6m – 36y)	Wechsler Intelligence Scale for Children (WISC), German Adaptation.	Case 1: at 6 years, WISC IQ = 100 on verbal subtests and WISC IQ = 85 on non-verbal subtests. Case 2: making good-average progress in a normal primary school. Case 3: received special support and had been “slow” in elementary school. Graduated basic secondary school.	7.5

*Demographic data were only presented for all participants within the study. Not all participants completed the cognitive assessments but the study does not report which of the participants took part.

**7 participants had a confirmed genetic diagnosis of Sotos syndrome.

***All participants had a confirmed genetic diagnosis of Sotos syndrome.

**Table 2:** Summary of studies measuring language abilities and/or specific cognitive abilities in Sotos syndrome (n = 13).

Author, country of study, year of publication	Sample size (n)	Gender	Mean age in years, months (range)	Cognitive assessment	Findings	Quality score (0–10)
Ball et al., USA, (2005)	16**	Not recorded*	6y 3m (1y 5m – 12y 3m)	Buffalo III Voice Screening Profile; Clinical Evaluation of Language Fundamentals Three Screening Test; Goldman-Fristoe Test of Articulation 2; Kahn-Lewis Phonological Analysis 2; Mean Length of Utterance in Morphemes; Peabody Picture Vocabulary Test (III); Preschool Language Scale 3; Social Skills Rating System; Type-token Ratio; Index of Augmented Speech Comprehensibility in Children.	Participants exhibited expressive and receptive language impairments, articulation impairments, voice impairments and stuttering.	8.2
Cole & Hughes, UK, (1994)	41	Not recorded*	Not recorded*	Parental recall.	Early delays in speech and performance skills. Older children had particular difficulties with short term memory, abstract ideas and practical reasoning. Numeracy was reported as the weakest area in older children, regardless of IQ.	5.6
Fickie et al., USA, (2011)	1***	1 (F)	63y	Wechsler Adult Intelligence Scale (WAIS-III).	The patient’s strengths were in verbal comprehension and behavioural regulation. Areas of weakness included working memory, interpretation of nonverbal information and processing speed.	8.3
Finegan et al., UK, (1994)	27	14 (M), 13 (F)	9y 3m (5y – 16y)	British Picture Vocabulary Scale Long Form; Expressive One-Word Picture Vocabulary Test, Upper Extension; Test for the Reception of Grammar; Word Structure Subtest of Clinical Evaluation of Language Fundamentals-Revised.	Language abilities were consistent with FSIQ scores. No relative deficits observed in language expression or comprehension. No significant difference in language abilities of Sotos group and comparison group when IQ was controlled.	9.5
Livingood & Borengasser, (1981)	1	1 (F)	1y 11m	Bayley Scales of Infant Development; Alpern-Boll Developmental Profile.	Bayley scales reflected mental functioning at the 15 month level and motor development at the 18 month level. Mother reported: physical age, self-help age and social age = 18 month level, academic age = 15 month level and communication age = 10 month level.	8.3
Mauceri et al., Italy, (2000)	4	3 (M), 1 (F)	(2y – 12y)	Brunet-Lezine Test; Clinical Observation.	Case 1: poor repetitive and expressive language. Case 3: delayed language acquisition. Case 4: mild delay in language. Case 6: severe deficit in language.	6.7
Morrow et al., USA, (1990)	1	1 (M)	4y 11m	Stanford Binet Intelligence Scale.	Stanford Binet scores: verbal reasoning = 96, abstract/visual = 94; quantitative reasoning = 98, short-term memory = 88. Exhibited both immediate and delayed echolalia.	7.5
Mouridsen & Hansen, Denmark, (2002)	1	1 (M)	3y 4m	Bayley Scales of Infant Development; Reynell Developmental Language Scales.	Expressive language was at 12–18 months level.	5.8
Park et al., Korea, (2014)	2***	2 (F)	(9m – 32y)	Bayley Scales of Infant and Toddler Development.	Case 1: 2 month delay in receptive language, 6 month delay in expressive language. Cognitive development delayed by 2 months. Case 2: normal intelligence but difficulty with expressive language.	7.5
Scarpa et al., Italy, (1994)	2	1 (M), 1 (F)	(5y – 7y)	Brunet-Lezine Test.	Case 1: delayed expressive language. Case 2: persistent language deficit.	5.8
Sotos et al., USA, (1964)	3	2 (M), 1 (F)	(7y – 11y 6m)	Clinical Observation.	Case 1: failed to speak until 3 years of age. Case 2: did not speak until 3 years of age. Case 3: immature speech.	7.5
Varley & Crnic, USA, (1984)	11	6 (M), 5 (F)	9y 5m (5y 11m – 13y 11m)	Wechsler Intelligence Scale for Children-Revised (WISC-R); Stanford Binet Intelligence Scale; Bayley Mental Scale.	Specific cognitive difficulties observed in language processing, attention span, concentration and visual-perceptual skills.	8.1
Zechner et al., Germany, (2009)	1***	1 (M)	10y 8m	Clinical Observation.	Expressive language delay.	7.5

*Demographic data were only presented for all participants within the study. Not all participants completed the cognitive assessments but the study does not report which of the participants took part.

**3 participants had a confirmed genetic diagnosis of Sotos syndrome.

***All participants had a confirmed genetic diagnosis of Sotos syndrome.

**Table 3:** Summary of studies measuring aggression and tantrums in Sotos syndrome (n = 6).

Author, country of study, year of publication	Sample size (n)	Gender	Mean age in years, months (range)	Cognitive assessment	Findings	Quality score (0–10)
Compton et al., USA, (2004)	1	1 (M)	20y	Psychiatric Assessment.	Admitted to an inpatient psychiatric facility due to the onset of psychotic symptoms (delusions and hallucinations). Parents reported a long history of angry outbursts and tantrums. Had received counselling for angry outbursts since 4 years of age. Quality of thinking was consistent with an underlying thought disorder.	7.5
Gajre et al., India, (2014)	1	1 (M)	11y	Parental and Teacher Rating NICHQ Vanderbilt Assessment Scales.	Behavioural problems included temper tantrums.	4.2
Gomes-Silva et al., Brazil, (2006)	1	1 (M)	3y 7m	Parental Report.	The mother reported that the patient had behaviour problems and was aggressive.	5
Mauceri et al., Italy, (2000)	3	3 (M)	(2y – 8y)	Parental Report; Teacher Report.	Case 1: parents observed behavioural problems. Poor social behaviour and aggressiveness was triggered when he was contradicted. Demonstrated pyromania. Case 2: teachers reported that he was aggressive towards the other children. Case 3: demonstrated aggressiveness.	6.7
Rutter & Cole, UK, (1991)	16	9 (M), 7 (F)	9y 4m (5y 11m – 14y 9m)	Rutter Questionnaires; Semi-Structured Interview with Parent.	On the Parent Questionnaire, scores ranged from 2–42 with a mean of 20.4. A Teacher Questionnaire was completed for 14 of the children. Scores ranged from 2–23 with a mean of 8.7. Parents reported that 13 of the children had problems with tantrums, 11 had sleep problems, 5 displayed precocious sexual behaviour, 10 had some form of phobia, 8 displayed ritualistic behaviour and 8 were obsessive about routines.	6.8
Trad et al., USA, (1991)	1	1 (F)	3y 11m	Psychiatric Assessment; Social Worker Report; DSM-III-R.	Her social worker noted that she displayed emotional impairment and either played alone or was aggressive with other children.	6.7

**Table 4:** Summary of studies measuring autistic features in Sotos syndrome (n = 4).

Author, country of study, year of publication	Sample size (n)	Gender	Mean age in years, months (range)	Cognitive assessment	Findings	Quality score (0–10)
Morrow et al., USA, (1990)	1	1 (M)	4y 11m	Clinical Observation.	Behaviour was characterised by repetitive and stereotypic head-banging and hair-pulling. Had previously demonstrated repetitive stroking of objects. Impairment in ability to interact socially. Authors report that the patient meets criteria for ASD.	7.5
Mouridsen & Hansen, Denmark, (2002)	1	1 (M)	3y 4m	ICD-10; Clinical Observation.	Case 1: met the ICD-10 diagnostic criteria for childhood autism. Showed repetitive and stereotypic behaviours as well as severe difficulties with reciprocal social interaction.	5.8
Trad et al., USA, (1991)	1	1 (F)	3y 11m	Psychiatric Assessment; Social Worker Report; DSM-III-R.	Met DSM-III-R criteria for Pervasive Developmental Disorder (PDD).	6.7
Zappella, Italy, (1990)	12	11 (M), 1 (F)	6y 9m (3y – 12y)	Behavioural Observation.	5 participants showed marked autistic behaviour.	7.5

**Table 5:** Summary of studies measuring attention deficit hyperactivity disorder (ADHD) in Sotos syndrome (n = 8).

Author, country of study, year of publication	Sample size (n)	Gender	Mean age in years, months (range)	Cognitive assessment	Findings	Quality score (0–10)
de Boer et al., Netherlands, (2006)	28**	Not recorded*	Not recorded*	Child Behaviour Checklist (CBCL); Young Adult Behaviour Checklist (YABCL); 18-item Dutch ADHD list; Dutch Questionnaire Derived from the American Parent and Teacher Questionnaire; Vineland Screener.	4 participants completed the CBCL (2–3 years). Of these, 1 scored in the clinical range for internalising behaviour problems. 19 completed the CBCL (4–18 years). Mean scores for total problems, internalising and externalising scales were significantly higher than the mean score for normative data. 5 participants completed the YABCL. Of these, 2 scored in the clinical range for total problems. 20 participants completed the ADHD-list. Mean scores of the whole group were not significantly different from the scores of the control group. 21 participants completed the Vineland Screener. Mean developmental ages were 1y 7m, 1y 7m and 2y 7m lower than the mean chronological ages for communication, daily living skills and social competence, respectively.	8.6
Finegan et al., UK, (1994)	27	14 (M), 13 (F)	9y 3m (5y – 16y)	Child Behaviour Checklist (CBCL); Teacher Report Form; Aberrant Behaviour Checklist; ADHD Rating Scale.	CBCL total scores were in the clinical range for 18 of the children by parent report and 17 by teacher report. Parents reported 10 participants as having ADHD.	9.5
Gajre et al., India, (2014)	1	1 (M)	11y	DSM V; Parental and Teacher Rating NICHQ Vanderbilt Assessment Scales.	Behavioural problems included inattention, hyperactivity and impulsiveness. Behavioural assessment led to a diagnosis of ADHD. Received behaviour modification therapy.	4.2
Gosalakkal, UK, (2004)	1	1 (M)	8y	Neuropsychological Evaluation.	Previously been diagnosed with ADHD. Current evaluation suggests possible ADHD and difficulty with impulse control.	1.7
Mauceri et al., Italy, (2000)	3	3 (M)	(2y – 12y)	Parental Report; Teacher Report.	Case 2: teachers reported that he was inattentive and hyperactive. Case 3: had a diagnosis of ADHD. Case 4: had a diagnosis of ADHD.	6.7
Mouridsen & Hansen, Denmark, (2002)	1	1 (M)	13y	ICD-10; Clinical Observation.	Case 2: attended a special education program for children with ADHD and later attended a class for children with autistic features. He was inattentive, hyperactive and difficult to manage.	5.8
Trad et al., USA, (1991)	1	1 (F)	3y 11m	Psychiatric Assessment; Social Worker Report; DSM-III-R.	Demonstrated lack of inhibition and impulsive behaviour.	6.7
Varley & Crnic, USA, (1984)	11	6 (M), 5 (F)	9y 5m (5y 11m – 13y 11m)	Psychiatric Evaluation; Achenbach Revised Child Behaviour Profile.	All participants had socialisation deficits. 9 met criteria for a psychiatric disorder. Of these, 3 had ADHD and 2 had organic personality syndrome. The scales most frequently elevated on the Achenbach Child Behaviour Profile were hyperactivity (n = 7), withdrawn/schizoid (n = 6), somatic complaints (n = 3) and obsessive (n = 3).	8.1

*Demographic data were only presented for all participants within the study. Not all participants completed the behavioural assessments but the study does not report which of the participants took part.

**11 participants had a confirmed genetic diagnosis of Sotos syndrome.

**Table 6:** Summary of studies measuring anxiety in Sotos syndrome (n = 2).

Author, country of study, year of publication	Sample size (n)	Gender	Mean age in years, months (range)	Cognitive assessment	Findings	Quality score (0–10)
Rutter & Cole, UK, (1991)	16	9 (M), 7 (F)	9y 4m (5y 11m – 14y 9m)	Rutter Questionnaires; Semi-Structured Interview with Parent.	Parents reported that 10 of the children had some form of phobia, 8 displayed ritualistic behaviour and 8 were obsessive about routines.	6.8
Sarimski, Germany, (2003)	27	17 (M), 10 (F)	10y 7m (6y – 15y)	Parental Report; Heidelerger-Kompetenz-Inventar (HKI); Children’s Social Behaviour Questionnaire (CSBQ); Nisonger Child Behaviour Rating Form (NCBRF).	According to the CSBQ, participants showed significantly more separation anxiety (*p* = .005) and tended to be more anxious (*p* = .08), compared to a control group of children with intellectual disabilities matched for age and cognitive ability. Participants with Sotos syndrome had higher scores in insecure/anxious behaviour (*p* < .05) compared to the control group.	9.5

### Quality Assessment

A quality checklist [19] was used to assess the quality of the studies included in this review. This checklist was chosen as it was designed specifically for use with quantitative studies, of various methodological designs. It has been used to assess the quality of papers included in a number of systematic reviews (e.g. [21,22]). A scoring manual provides detailed guidelines for assessing the quality of the research. The checklist was used in its original form, though questions 5–7 (from the original checklist) were removed as they related to intervention studies, so were not relevant for this review (see S2 Appendix). The quality of all of the papers included in the review was assessed in relation to the topic of interest (cognition or behaviour), as opposed to the quality of the paper in general. The scores were rated out of 10.

## Results

**Fig 1:**
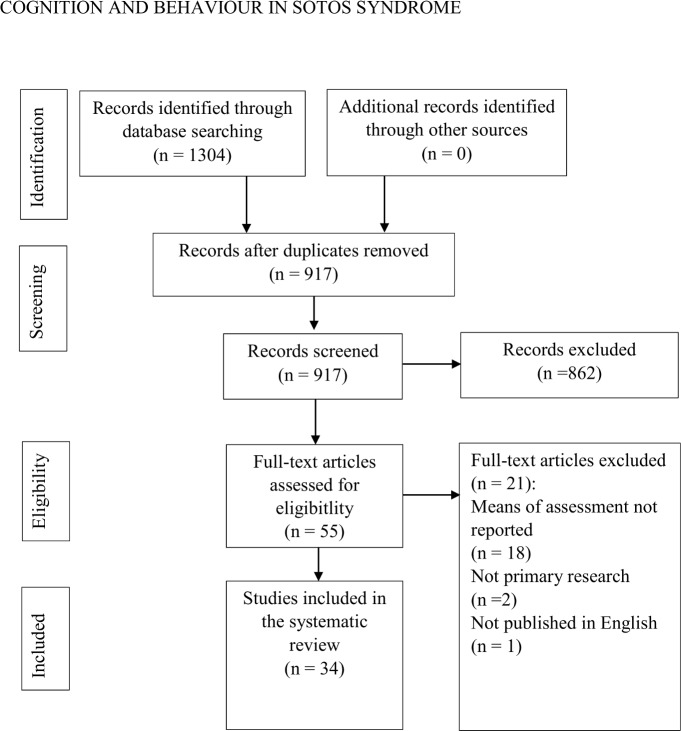
Search strategy and study inclusion (August 2015).

The literature search yielded 1304 results. Once duplicate results had been removed, a total of 917 articles were screened for inclusion in the review. The abstracts of these papers were read and papers were considered to be relevant if the abstract met all inclusion criteria. After the abstracts had been screened, fifty five full articles were read to assess eligibility for the review. Eighteen articles were excluded on the basis that the means of assessment for cognitive or behavioural data were not reported, two were excluded because no primary research was reported and one was excluded due to not being published in English. As a result, a total of thirty four articles met inclusion criteria (see Fig 1). Crucially, the search revealed that no systematic reviews or meta-analyses have been published in the Sotos literature.

### Quality of Included Studies

Each article was assessed by the first author in order to establish the quality of the research. The articles were assessed in relation to specific criteria based on objectives, methodology, results and conclusions. The score for each article is provided in Tables 1–6. A second reviewer independently assessed the quality of 20% of the studies in order to ensure that the assessment was reliable. Intraclass correlation coefficient for the two reviewers was .86, indicating excellent inter-rater reliability [23]. Both of the reviewers ranked the papers in the same order (lowest-highest). The mean score was 6.8 (*SD* = 1.67) and scores ranged from 1.7–9.5. This highlights that there is considerable variation within the quality of the published literature, providing data on cognition and/or behaviour in Sotos syndrome.

### Common Themes Emerging from Study Findings

A small number of studies (n = 10) have used a group study design to assess cognitive and/or behavioural features of individuals with Sotos syndrome. The use of cohorts of individuals has allowed comparisons to be made between participants, providing an insight into common cognitive and behavioural phenotypes. A case study design was implemented in more than half of the studies (n = 24). This means that a significant proportion of the data reported in relation to cognition and behaviour in individuals with Sotos were based on very small samples. The use of case study design makes it is difficult to establish whether there is a consistent cognitive or behavioural pattern associated with the syndrome as the findings often lack generalisability. However, data from case studies are useful in providing a detailed analysis of cognition and behaviour in individuals with Sotos.

### Intelligence quotient (IQ)

Cognitive abilities were assessed, using standardised measures of IQ, in a total of 172 participants, across twenty five studies (see Table 1). Of these, six were group studies and nineteen were case studies. The most common measures of IQ were versions of the WISC (used in eleven studies) and the Stanford Binet Intelligence Scale (used in eight studies). In two group studies, a mean full scale IQ (FSIQ) of all the Sotos participants included in the study was reported. These were 76 [24] and 73.8 [25]. The number of participants in each of these studies was 21 and 15, respectively. Varley & Crnic [26] reported a median FSIQ of 62 for the 11 participants included in this study. A limitation of the remaining three group studies is that the mean or median FSIQ was not reported [27–29]. In one study [29], cognitive abilities were assessed in terms of cognitive competence so findings from this study are not comparable with the other group studies that measured FSIQ.

Of the six group studies that reported FSIQ scores, four reported the range of these scores. These were 47–105 [24], 21–103 [27], 54–96 [25] and 40–85 [26]. This shows that there is a consistent range of ability reported in all of the studies that provided the range of FSIQ scores, suggesting that individuals with Sotos syndrome can be higher functioning, though most are not. In general, the literature suggests that the majority of individuals with Sotos syndrome have mild intellectual disability (IQ = 50–69) or are in the borderline range (IQ = 70–84). However, level of intellectual functioning is variable and a few cases of severe intellectual disability or intellectual ability within the normal range have been reported.

In addition to FSIQ scores, seven studies [24,25,30–34] also reported performance IQ and verbal IQ scores. This information provides an insight into ability in the two separate domains that comprise FSIQ. Verbal IQ scores were reported to be higher than performance IQ scores in all studies, except one case study [34]. However, in this study, the participant was reported to have a performance IQ of 101 and a verbal IQ of 100. Overall, the evidence suggests that individuals with Sotos syndrome have better verbal IQ, compared to performance IQ scores.

Other than reporting performance IQ and verbal IQ scores, only one study [35] reported quantitative scores in four specific cognitive domains (verbal reasoning, abstract/visual, quantitative reasoning and short-term memory). This was a case study, reporting findings relating to a 4y 11m old male. As data were based on one young child, it provides only a limited insight into the cognitive profile of individuals with Sotos. Specific areas of cognitive ability and/or disability were reported in three other studies [4,26,36]. All of the studies reported non-verbal reasoning as a particular area of weakness. However, the degree of ability in the specific areas that were mentioned in each of the studies was not reported in a quantitative format. As a result it is difficult to compare whether participants from each of these studies were performing at a similar ability level and the extent to which the abilities in specific cognitive domains deviated from the general ability of each participant.

In summary, the focus of previous research has been to investigate degree of intellectual functioning in individuals with Sotos syndrome. This has identified that the majority of individuals with Sotos syndrome have intellectual disability (IQ <70) or are in the borderline range (IQ = 70–84). In addition, the profile of intellectual functioning suggests that individuals achieve higher verbal IQ scores, compared to performance IQ scores. At present, only one case study [35] has reported quantitative scores on specific cognitive sub-scales.

### Language

Language abilities were reported in thirteen studies (see Table 2). Finegan et al. [27] used the largest sample (n = 27) to assess language abilities using various standardised language assessments. Language abilities were examined in relation to general intellectual ability, as opposed to assessing absolute language impairment. The findings from this study indicated that language abilities were consistent with FSIQ scores and that participants exhibited no relative deficits in language comprehension or expression, when compared to general level of intellectual functioning. In this study, language abilities were compared to a control group matched for IQ and no significant difference between language impairment in the two groups was identified. Consequently, it is important to consider language development in relation to general intellectual development in order to establish whether specific language impairments are associated with Sotos syndrome. This study scored 9.5 on the quality checklist and the research is therefore of a high standard. The findings from this study support the argument that individuals with Sotos have better verbal IQ, compared to performance IQ. A replication of this finding would improve the reliability of the argument that Sotos syndrome is not associated with absolute language deficits.

In contrast, other research has found that individuals with Sotos display relative language impairment. Delays in speech and communication were reported in four studies [2,4,37,38], indicating that speech and communication is delayed, when compared to language development in typically developing children. However, as level of intellectual functioning was not reported, it is difficult to establish whether delays were relative or absolute. Ball, Sullivan, Dulany & Schaefer [39] found that participants had both expressive and receptive language impairments. However, Mouridsen & Hansen [33], Scarpa, Fraggioloi & Voghenzi [40] and Zechner et al. [41] reported delays in expressive but not receptive language. Park, Lee, Sohn & Ko [42], reported cases of a mother and her 9 month old daughter with Sotos syndrome. The daughter was reported as having both receptive and expressive language difficulties whereas the mother only showed difficulty with expressive language. In all of these studies, language abilities were not compared to a control group matched for intellectual functioning and were often based on clinical observation. It is therefore difficult to establish whether relative language impairment is specific to the Sotos population or whether it is a consequence of the associated intellectual disability and developmental delay.

Although speech and language delays have been reported in eleven of the thirteen studies that assessed language abilities, two studies [27,36] did not report relative language impairment. Language abilities were assessed using a comprehensive battery of language assessments in one study [27] and the findings from this study suggest that individuals with Sotos display language abilities that are consistent with their general level of intellectual functioning. In addition, findings from the remaining studies that investigated language abilities indicate that individuals with Sotos may display speech and language delays, when compared to typically developing controls. Specifically, individuals with Sotos appear to experience greater difficulty with expressive, compared to receptive language.

### Aggression and tantrums

Aggressive behaviour and/or tantrums were reported in six studies [25,31,38,43–45] and were assessed through parental report or psychiatric assessment (see Table 3). Of these studies, five employed a case study design and only one of the case studies used a female participant [45], despite the syndrome affecting males and females equally. In the group study [25], parents were asked to describe the behavioural and emotional problems experienced by their child. Thirteen of the sixteen participants were described as having tantrums in the home environment. However, participants may have come to medical attention as a result of behavioural issues so this sample may not be representative of the Sotos population.

It is important to note that all of the participants reported to have these behavioural issues were children. Consequently, no research has investigated whether these behavioural issues persist during adulthood. As children with Sotos are often large for their age, behavioural issues may be considered more problematic by others when the child is compared to another child of similar age and/or size. None of the studies used a control group so it is difficult to establish whether children with Sotos display significantly more aggressive behaviour and/or tantrums than other children of similar intellectual ability.

### Autistic features

Autistic features were reported in four studies (see Table 4). One study investigated behaviour in a group of twelve individuals with Sotos and reported autistic features in five of these participants [46]. Autistic features were assessed based on clinical observation. A clinical diagnosis of Autism Spectrum Disorder (ASD) was reported in two case studies [33,35] of young male participants (4y 11m and 3y 4m, respectively). In addition, Pervasive Developmental Disorder (PDD) was reported in a case study [45] of a young female participant (3y 11m). This suggests that ASD may be prevalent in individuals with Sotos syndrome. However, this has not been compared with prevalence of ASD within the intellectual disabilities population and no systematic study in this area has yet been conducted.

### Attention deficit hyperactivity disorder (ADHD)

Of the group studies that assessed behaviour, two reported a high prevalence of ADHD (see Table 5). Finegan et al. [27] found that 10 of the total 27 participants had ADHD (as measured by parental report) and Varley & Crnic [26] found that 3 of the total 11 participants met diagnostic criteria for ADHD. However, deBoer et al. [24] found no significant difference between mean scores of the Sotos group and the control group, on the 18-item Dutch ADHD list. Within the case studies that measured behavioural features of individuals with Sotos syndrome, a total of five participants were reported to have a clinical diagnosis of ADHD [33,38,43,47]. In addition, two cases were reported of individuals who were inattentive, hyperactive and demonstrated a lack of inhibition [33,45]. Findings from these studies suggest that ADHD may be a common behavioural problem associated with Sotos syndrome, though no systematic study in this area has yet been conducted.

### Anxiety

Anxiety has been reported in two studies (see Table 6). Sarimski [29] measured anxiety using The Children’s Social Behaviour Questionnaire (CBSQ) and found that children with Sotos displayed significantly more separation anxiety and had a tendency to be more anxious in new situations when compared to a control group matched for age and cognitive ability. Furthermore, the Sotos group had higher scores in insecure/anxious behaviour (as measured by the Nisonger Child Behaviour Rating Form (NCBRF)), when compared to the matched control group. In addition, Rutter & Cole [25] found that ten of the total sixteen participants had some form of phobia, as described through parental report. This suggests that anxious behaviour may be more prevalent within the Sotos population, compared to children with intellectual disabilities. There may also be a specific profile of anxious behaviour in individuals with Sotos but this needs to be investigated in further research.

### Longitudinal studies

One of the cardinal features of Sotos syndrome is developmental delay and children with the syndrome may follow a distinct developmental trajectory. In order to identify the progression of cognitive development in individuals with Sotos, it is important to investigate developmental changes, over time. One study [48] provided longitudinal data for a small number of participants (n = 10). To date, this is the only published longitudinal study that has reported data relating to cognitive abilities in individuals with Sotos syndrome. Cognitive tests were administered to all participants and eight of these were also assessed in at least one-follow up session. The age at which participants were assessed ranged from 1y – 13y 6m. Broadly, the study found that intellectual abilities improved with age and that IQ scores were in the range of 56–113. Each participant was administered different cognitive assessments at various ages so it is difficult to establish whether a consistent pattern of cognitive abilities exists in this population.

### Participants

Within the thirty four studies that were included in this review, cognitive abilities and/or behavioural features were reported for a total of 247 participants. Of the studies that reported group data, none of the participants were adults. Cognitive and/or behavioural data were presented in seven case reports of adults with Sotos [30–32,36,41,42,49]. The fact that there is such a small amount of data relating to cognition in adults with Sotos means that it is difficult to establish whether there is a specific profile or trajectory of cognitive ability associated with the syndrome.

Less than half of the studies (n = 14) were published after identification of the genetic abnormality associated with Sotos syndrome. Of these studies, eight [24,36,39,41,42,49–51] reported the number of participants with a confirmed genetic diagnosis of Sotos syndrome.

## Discussion

The primary of this review was to synthesise and critically evaluate all published literature providing data on cognition and behaviour in individuals with Sotos syndrome in order to establish current understanding of these facets of the syndrome. The specific research questions were to establish: 1) the degree of intellectual disability in individuals with Sotos syndrome; 2) whether there is evidence for a profile of verbal and non-verbal cognitive abilities; 3) whether there are common behavioural problems associated with Sotos, such as psychiatric problems and issues with temperament. The quality of the identified research was assessed using a standardised checklist and scores were rated out of 10. The mean score was 6.8 (*SD* = 1.69) and scores ranged from 1.7–9.5. The findings from the published literature were extracted and summarised in order to provide a comprehensive overview of current understanding of cognition and behaviour in Sotos syndrome.

Broadly, the literature suggests that the majority of individuals with Sotos syndrome have mild intellectual disability (IQ = 50–69) or are in the borderline range (IQ = 70–84) and this evidence supports the inclusion of intellectual disability as one of the main diagnostic criteria of the syndrome. In addition, findings from research using intelligence tests indicate that verbal IQ scores are consistently higher than performance IQ scores. Language abilities are relative to general level of intellectual functioning [27]. Language delays are more commonly reported in expressive, compared to receptive language [33,40,42]. Behavioural problems that may be common in Sotos syndrome are ASD [35,46], ADHD [26,27], anxiety [29] and aggression/tantrums [25,31]. However, no systematic study has been conducted in relation to these behavioural issues so it is difficult to establish whether there is a specific behavioural profile associated with Sotos syndrome. In addition, prevalence of behavioural problems has not been compared to prevalence within a sample of individuals of a similar intellectual ability.

The cognitive literature identified that almost all of the reported cases of Sotos syndrome have a degree of intellectual disability. This ranged from mild to severe. The International Statistical Classification of Diseases and Related Health Problems (ICD-10) suggests the following guidelines for classification of the degree of intellectual impairment: borderline intellectual functioning (70–84), mild intellectual disability (IQ = 50–69), moderate intellectual disability (IQ = 35 and 49) and severe intellectual disability (IQ = 20–34). Most of the cognitive data were presented in the form of an IQ score and the research to date has focused on the use of intelligence tests to measure cognition. The informative value of a full scale IQ score alone is limited in terms of its contribution to identifying ability in specific cognitive domains. Although this can provide a general indication of intellectual ability, it does not provide any information relating to strengths or weaknesses in different aspects of cognition. Thus, in order to establish whether individuals diagnosed with Sotos syndrome have a consistent cognitive profile, it will be necessary to investigate patterns of ability and disability in specific cognitive domains using a standardised battery of cognitive tests.

Behavioural problems such as aggression/tantrums, ASD, ADHD and anxiety have been reported in fourteen studies of individuals with Sotos syndrome. More than half of these were cases studies and as a result, the findings within the behavioural literature are based on a limited sample size. It has been suggested that children with Sotos may display more behavioural problems, compared to typically developing children [4,29]. This could be due to the fact that children with Sotos are usually large for their age and are therefore often mistaken as older and more able than their actual developmental level. This assumption can lead to frustration for the child which then manifests itself in behavioural problems. In order to determine whether behavioural problems are syndrome-specific, it is essential for behavioural features to be assessed in a representative sample and for findings to be compared with a matched control group.

This review only included published studies as an aim of the review was to establish the current understanding of literature reporting data on cognition and behaviour in Sotos syndrome. It is important to note that a limitation of this approach is that the review is subject to publication bias. In addition, only papers published in English language were reviewed which means that findings from data published in other languages were automatically excluded from the review.

### Limitations of Reviewed Studies

More than half of the studies included in this review were published prior to identification of the NSD1 genetic abnormality which was identified in 2002 [7]. It is therefore not possible to ascertain how many of the participants were NSD1-positive. This means that it is difficult to compare the cognitive and behavioural phenotypes of individuals with or without the genetic abnormality. Tatton-Brown et al. [18] investigated 239 cases of Sotos syndrome with NSD1 mutations. This study provided a detailed understanding of the clinical phenotype associated with NSD1-positive individuals. However, the main aim of this research was to investigate the whole clinical phenotype (facial dysmorphism, childhood overgrowth, scoliosis etc.) so cognition and behaviour were not explored in detail. DeBoer et al. [24] investigated IQ scores in both NSD1 mutation (n = 12) and NSD1 non-mutation (n = 17) participants and found that there was no significant difference between the IQ scores of these two groups of participants. However, in this study, two of the participants were considered to be too young to take part and six were excluded on the basis that they were uncooperative. Therefore future research should look to investigate cognition and behaviour in a larger and more representative sample of NSD1 mutation and NSD1 non-mutation participants, in order to establish a more detailed understanding of the cognitive and behavioural profiles of these individuals.

As stated by Cole & Hughes [4], a number of patients reported within the literature have come to medical attention due to developmental delay. Consequently, this may have resulted in a bias for recruitment of participants with more severe intellectual disability and/or behavioural problems. As awareness of Sotos syndrome is fairly limited, this is a difficult issue to overcome. Any individuals who do not present with significant symptoms or who are not assessed by a clinician who is aware of the syndrome, are less likely to be given a diagnosis of Sotos syndrome. Thus, until there is greater awareness of the syndrome, it will be difficult to assess cognitive and behavioural facets in a large and fully representative sample.

A fundamental methodological issue present in most of the studies included in this review is the limited sample size. As Sotos syndrome has a relatively low incidence, there is a limited population from which to recruit participants. It is therefore important for future research to utilise all available recruitment strategies in order to collect a large and representative data set. A further methodological problem, identified in more than half of the studies, was a failure to use standardised measures to assess cognition and/or behaviour or, in some cases, a failure to report which measures were used. Findings from these studies lack validity as it is not clear whether the results were obtained using standardised measures. As a result, these studies tended to score lower on the quality assessment checklist.

### Directions for Future Research

A number of cognitive and behavioural features have been identified in individuals with Sotos syndrome such as language difficulties [39], ADHD [26] and ASD [46]. However, these are based on limited samples. It is therefore essential for future research to explore these facets in a representative, population sample, using the same standardised measures for all participants. Data from a matched control group could be compared with data from individuals with Sotos in order to determine whether there are significant differences in the rate of cognitive development and the age at which behavioural problems arise. In addition, research with adults would enhance current understanding of the trajectory of cognitive development in Sotos syndrome, an area in which there is currently very little published research.

The suggestion that verbal IQ scores are higher than performance IQ scores in Sotos is particularly interesting as the opposite is often reported in individuals with ASD [52,53]. As ASD has been reported in some individuals with Sotos, future research could investigate the direction of the discrepancy between verbal IQ and performance IQ in individuals with a diagnosis of Sotos who have high levels of autistic traits, or even a comorbid diagnosis of ASD. In addition, the suggestion that ASD may be linked to Sotos is based on limited data and therefore, future research should investigate co-morbidity in a larger sample.

Future research could combine genetic information with both cognitive and behavioural data, in order to establish a comprehensive understanding of genotype-phenotype correlations within this population. This could include individuals with NSD1 deletions or NSD1 mutations and individuals who are considered to be Sotos-like, who may have abnormalities of the NFIX protein.

Cognitive and behavioural phenotyping of disorders and syndromes associated with intellectual disability can be extremely beneficial for individuals. For example, research has established specific cognitive profiles associated with Williams syndrome [54] and Down’s syndrome [55]. Much of the literature included in this review has investigated intellectual functioning in Sotos syndrome, as opposed to focusing on specific cognitive abilities. The individual components, or subscales, that comprise general intelligence scores could be investigated in individuals with Sotos syndrome in order to establish whether there is a consistent pattern of ability and/or disability in individuals with Sotos syndrome. Specifically, a cognitive profile can inform education and allow appropriate teaching techniques to be implemented, in order to enhance learning and development. In addition, an awareness of associated behavioural, social and emotional problems can lead to quicker identification and the implementation of effective management strategies.

In summary, during the fifty one years since the initial recognition of Sotos syndrome, a total of thirty four papers reporting data on cognition and/or behaviour in Sotos syndrome have been published in peer-reviewed journals. The current literature supports the view that a significant number of individuals with Sotos syndrome have an associated intellectual disability (IQ < 70) and nearly all participants had an FSIQ score < 100. The highest reported FSIQ score was 113 [48] and the lowest was 21 [27], indicating significant variability in intellectual functioning within the Sotos population. Few studies have explored specific cognitive abilities but there is evidence to suggest that verbal IQ scores may be higher than performance IQ scores. Language abilities seem to be consistent with general level of intellectual functioning. Fourteen studies have provided data on behavioural features in Sotos syndrome and the findings suggest that there may be a high prevalence of ADHD, anxiety, aggression/tantrums and ASD within the Sotos population. Although a range of studies have provided insight into cognition and behaviour in individuals with Sotos, syndrome-specific cognitive and behavioural profiles have not yet been fully specified. This review provides an overview of current knowledge of cognition and behaviour in Sotos syndrome and suggests areas for future research.

## Supporting Information

S1 Appendix Quality Assessment Checklist (revised from Kmet, Lee & Cook, 2004). (DOCX)

S2 Appendix PRISMA Checklist. (DOCX)
