# Supplementary material for: Cognition and Behaviour in Sotos Syndrome: A Systematic Review
Source: PLoS One. 2016 Feb 12;11(2):e0149189. doi: 10.1371/journal.pone.0149189 (PMC4752321; doi:10.1371/journal.pone.0149189)
Supplement: S1 Appendix — (DOCX) [file pone.0149189.s001.docx]

**S2 Appendix. Quality Assessment Checklist (revised from Kmet, Lee & Cook, 2004).**

|  | **Criteria** | **Yes (2)** | **Partial**  **(1)** | **No (0)** | **N/A** |
| --- | --- | --- | --- | --- | --- |
| **1** | Question/objective sufficiently described? |  |  |  |  |
| **2** | Study design evident and appropriate? |  |  |  |  |
| **3** | Method of subject/comparison group selection or source of information/input variables described and appropriate? |  |  |  |  |
| **4** | Subject (and comparison group, if applicable) characteristics sufficiently described? |  |  |  |  |
| **5** | Outcome and (if applicable) exposure measure(s) well defined and robust to measurement/misclassification bias? Means of assessment reported? |  |  |  |  |
| **6** | Sample size appropriate? |  |  |  |  |
| **7** | Analytic methods described/justified and appropriate? |  |  |  |  |
| **8** | Some estimate of variance is reported for the main results? |  |  |  |  |
| **9** | Controlled for confounding? |  |  |  |  |
| **10** | Results reported in sufficient detail? |  |  |  |  |
| **11** | Conclusions supported by results? |  |  |  |  |
